# Toward Rapid, Widely Available Autologous CAR-T Cell Therapy – Artificial Intelligence and Automation Enabling the Smart Manufacturing Hospital

**DOI:** 10.3389/fmed.2022.913287

**Published:** 2022-06-06

**Authors:** Simon Hort, Laura Herbst, Niklas Bäckel, Frederik Erkens, Bastian Niessing, Maik Frye, Niels König, Ioannis Papantoniou, Michael Hudecek, John J. L. Jacobs, Robert H. Schmitt

**Affiliations:** ^1^Fraunhofer Institute for Production Technology IPT, Aachen, Germany; ^2^Institute of Chemical Engineering Sciences, Foundation for Research and Technology-Greece (FORTH), Patras, Greece; ^3^Skeletal Biology and Engineering Research Centre, Department of Development and Regeneration, KU Leuven, Leuven, Belgium; ^4^Prometheus the Leuven R&D Translational Division of Skeletal Tissue Engineering, KU Leuven, Leuven, Belgium; ^5^Lehrstuhl für Zelluläre Immuntherapie, Medizinische Klinik und Poliklinik II, Universitätsklinikum Würzburg, Würzburg, Germany; ^6^ORTEC BV, Zoetermeer, Netherlands; ^7^Laboratory for Machine Tools and Production Engineering (WZL) of RWTH Aachen University, Aachen, Germany

**Keywords:** ATMP, CAR-T cell, artificial intelligence, automation, autologous, manufacturing, Industry 4.0, smart manufacturing hospital

## Abstract

CAR-T cell therapy is a promising treatment for acute leukemia and lymphoma. CAR-T cell therapies take a pioneering role in autologous gene therapy with three EMA-approved products. However, the chance of clinical success remains relatively low as the applicability of CAR-T cell therapy suffers from long, labor-intensive manufacturing and a lack of comprehensive insight into the bioprocess. This leads to high manufacturing costs and limited clinical success, preventing the widespread use of CAR-T cell therapies. New manufacturing approaches are needed to lower costs to improve manufacturing capacity and shorten provision times. Semi-automated devices such as the Miltenyi Prodigy^®^ were developed to reduce hands-on production time. However, these devices are not equipped with the process analytical technology necessary to fully characterize and control the process. An automated AI-driven CAR-T cell manufacturing platform in smart manufacturing hospitals (SMH) is being developed to address these challenges. Automation will increase the cost-effectiveness and robustness of manufacturing. Using Artificial Intelligence (AI) to interpret the data collected on the platform will provide valuable process insights and drive decisions for process optimization. The smart integration of automated CAR-T cell manufacturing platforms into hospitals enables the independent manufacture of autologous CAR-T cell products. In this perspective, we will be discussing current challenges and opportunities of the patient-specific but highly automated, AI-enabled CAR-T cell manufacturing. A first automation concept will be shown, including a system architecture based on current Industry 4.0 approaches for AI integration.

## Introduction

**Figure 1:**
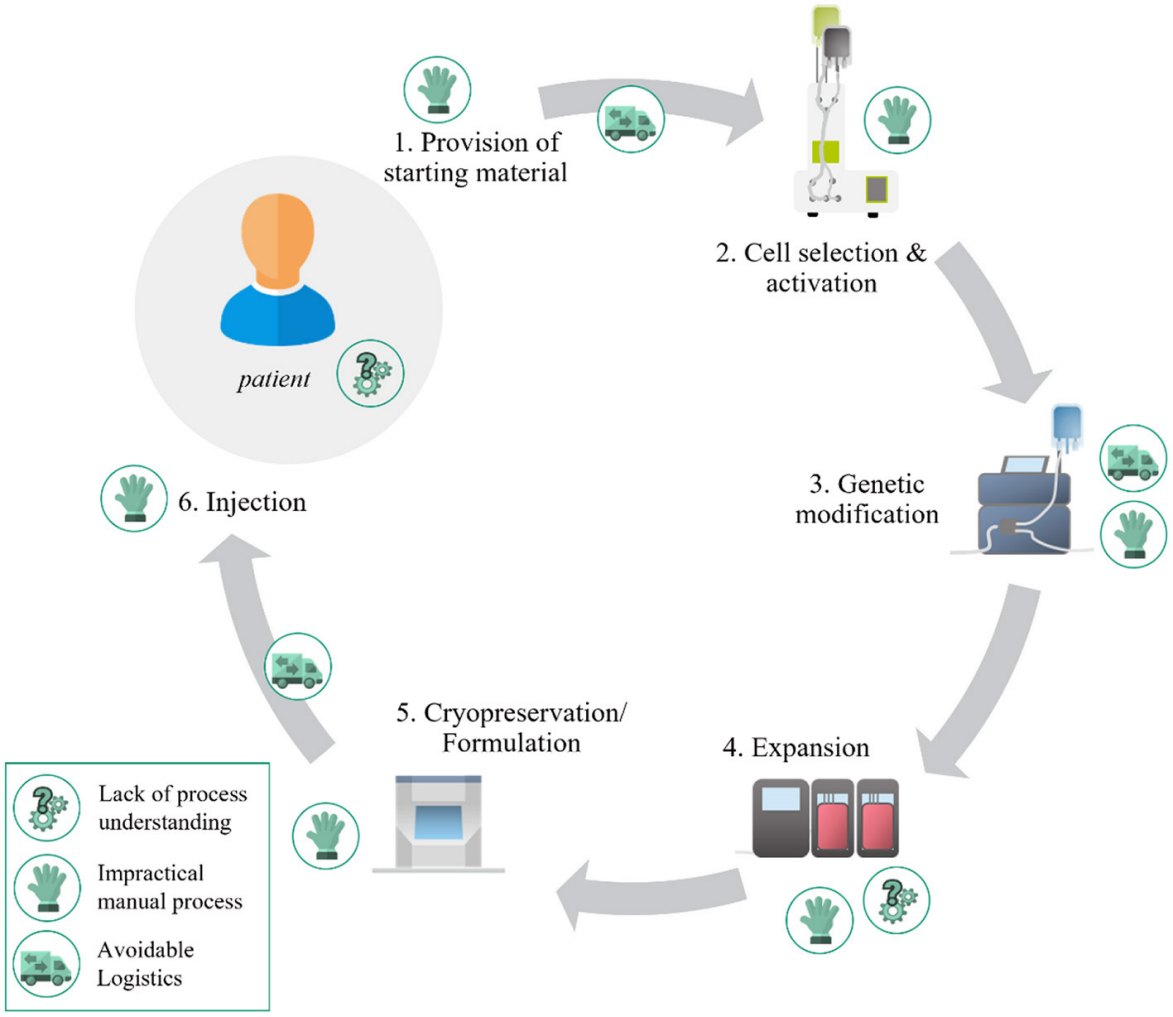
Challenges for wide-scale deployment of autologous CAR-T cell therapy.

With the transformation of hospitals toward smart treatment centers, digitalization is entering the health sector and supporting hospital employees through intuitive digital data management and robotics. Successes have already been achieved in diagnostics, treatments, and surgical intervention. In recent years, Advanced Therapy Medicinal Products (ATMPs) have gained importance for curing genetic and cellular diseases. One therapy already being applied to treat acute leukemia and lymphoma is CAR-T cell therapy. In contrast to traditional cancer treatments, CAR-T cell therapy allows for the specific targeting of tumor cells. The approved therapies Kymriah^®^, Yescarta^®^, and Tecartus^®^ (1–3) target the CD19 antigen in hematological malignancies but differ in cell composition, manufacturing process, and costimulatory domain. These therapies use an autologous approach, where the patient's cells are engineered instead of allogeneic cell therapies where cells are extracted from a healthy donor, engineered, and expanded to treat multiple other patients. Allogeneic CAR-T cells offer the opportunity for large-scale production. However, they cause significant graft-vs.-host disease and are rapidly terminated by the host's immune system, currently limiting their applicability (4). Autologous therapies have seen clinical approval but face manufacturing and large-scale deployment challenges. Figure 1 visualizes these challenges along the six main steps of CAR-T cell therapy.

The entire process from provision of starting material (e.g., apheresis, blood donation) to injection is currently dominated by **impractical manual processes**. These processes are highly complex, requiring much personnel and generating high costs due to their labor-intensiveness, cost of materials, and use of large cleanroom suites. Additionally, manual manufacturing leads to frequent interaction of personnel and product, increasing the risk for contamination and subsequent product loss. A transition away from these manual and static manufacturing protocols is needed to shorten production cycles to improve vein-to-vein timelines. As autologous therapies are keyed to an individual patient, the current centralized production increases overall manufacturing times and generates **avoidable logistics** due to laborious transportation of apheresis, viral vectors, and CAR-T cell product. Compared to the established therapies the vein-to-vein timelines [e.g., 17 days for Yescarta (5)] can be reduced and consequently the patient's chances of recovery increased.

Closed, semi-automated systems have been developed to address these issues, such as the Miltenyi Prodigy^®^ and the Lonza Cocoon^®^ (6, 7). These devices follow a “one-device-per-patient approach” to minimize the risk of cross-contamination. Unfortunately, this manufacturing approach is unsuited for large-scale deployment, limiting the reduction of manufacturing costs and widespread application of CAR-T cell therapy. These devices are time-consuming to adapt to technological advances in the field due to their high level of integration and technological complexity. Additionally, these devices do not provide the necessary process insights to assess cell quality and provide early information on the performance of the cells and potential therapeutic outcome.

Installing more process analytical technology to generate a broad data basis combined with data analytics and AI approaches is needed to overcome a **lack of process understanding**. Even if regulatory hurdles often still have to be overcome, AI is already being used successfully in the hospital context (8). McKinsey created an overview of the AI solutions currently used in Europe and classified them according to a patient-centered healthcare framework (9). Most of the identified use cases are in diagnostics and clinical decision making, whereas a typical use case in diagnostics is the automated counting of living and dead cells in a blood sample. CAR-T cell therapy can highly benefit from the solutions already in use and the overall potential of AI.

A novel automated manufacturing approach is needed to treat high numbers of patients with autologous CAR-T cell therapies at the state-of-the-art. This automated system should allow for parallelized production of autologous CAR-T cell products to decrease costs and increase the product's availability. It needs to be designed in an integrated but modular manner to allow for rapid adaption to technological advancements in the field of ATMP manufacturing but also decreases hands-on interventions to a minimum.

The automation and AI integration require the second transformation of hospitals into smart manufacturing hospitals by enabling them to produce CAR-T cells directly at the point of care. A **smart manufacturing hospital** is defined as a hospital specialized on ATMPs that incorporates an end-to-end automated manufacturing platform for personalized treatment in an adjacent GMP facility. The facility connects to existing logistic and IT infrastructure, offering extensive patient and manufacturing data availability as well as AI-driven clinical decision support while taking all regulations (e.g., G(A)MP, MDR, cybersecurity) into account. For a flexible and modular integration of the manufacturing platform into the GMP facility and the hospital, an IT infrastructure based on reliable Industry 4.0 and IIoT (Industrial Internet of Things) is needed to cope with the rapidly changing environment of automated cell and gene therapy. Existing approaches (10–12) already show applicability to the hospital context but do not meet the new demands of the smart manufacturing hospital. In particular, the reliable provision of patient and manufacturing data and comprehensible decision support for the manufacturing process requires a novel, holistic approach to exploit the full potential of automated CAR-T cell therapy.

## Automated AI-Driven CAR-T Cell Manufacturing

**Figure 2:**
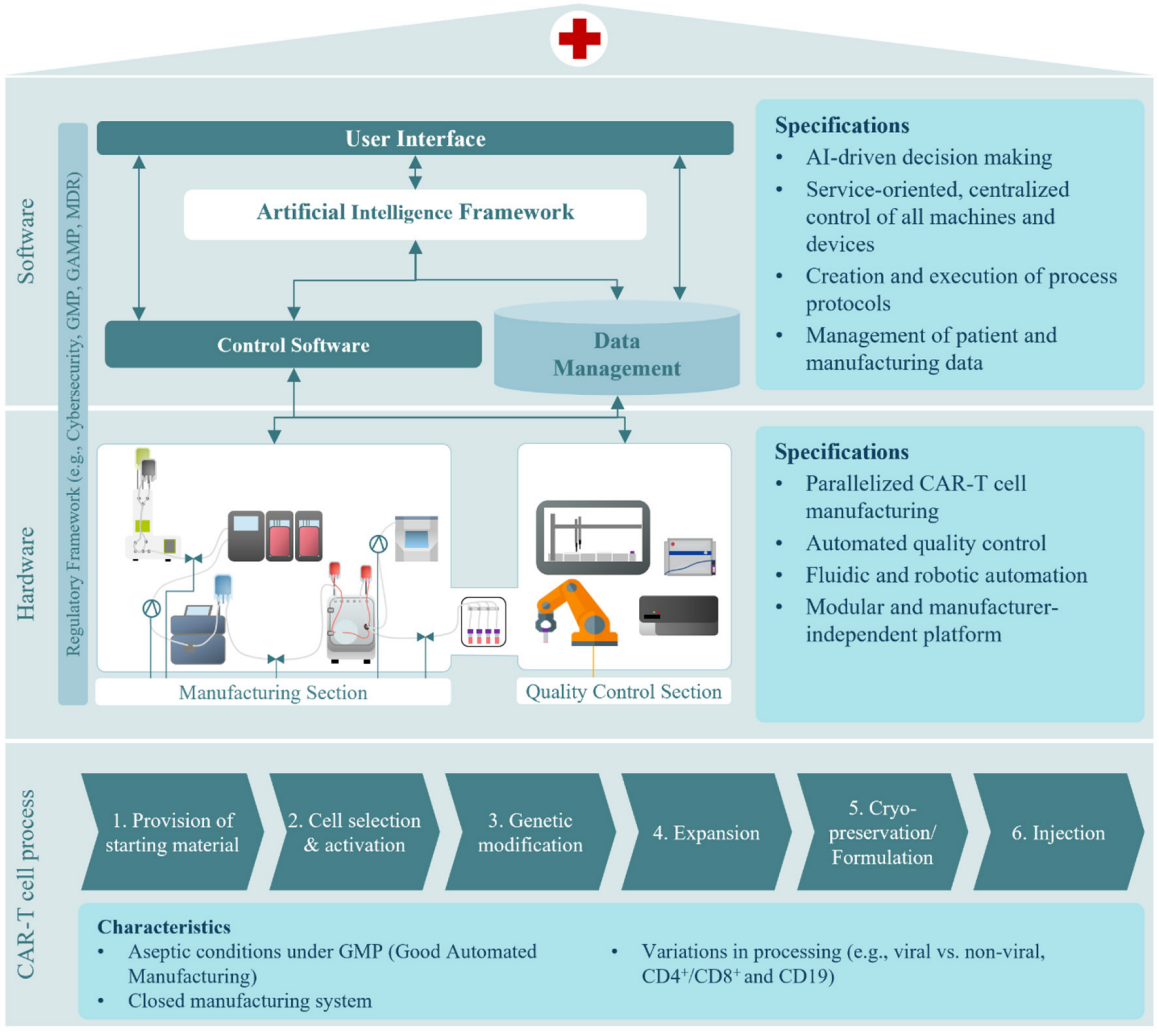
Automated, AI-driven CAR-T cell manufacturing concept considering the CAR-T cell process, hardware, and software components.

For decentralized onsite manufacturing of ATMPs, the hospital infrastructure needs to be adapted. As such, a manufacturing platform should operate mostly independent of highly trained personnel producing autologous CAR-T cells autonomously. The deployment of an automated AI-driven CAR-T cell manufacturing platform requires extensive knowledge of the underlying biological process, hardware (e.g., devices, machines), and software components (e.g., control software, data management, AI models). Since the three areas are highly interconnected, a close interdisciplinary exchange of all stakeholders is essential for success. Our automated AI-driven CAR-T manufacturing concept, developed within the scope of EU H2020 project AIDPATH (AI-driven, Decentralized Production for Advanced Therapies in the Hospital) (13, 14), focuses on these three areas and approaches the technological challenges and potential solutions. Figure 2 gives an overview of the manufacturing platform executing the CAR-T cell process and the software components enabling process control, AI integration, and data management.

The **CAR-T cell process** poses several challenges toward automation. Firstly, as an ATMP production process, it must be conducted under aseptic conditions and according to Good Manufacturing Practice (GMP) (15). Maintaining large cleanroom suites in a hospital for ATMP manufacturing is impractical, therefore aseptic production in cleanrooms with lower grade and smaller footprints is essential. Secondly, for sustained use of the automated manufacturing platform in CAR-T cell manufacture, it should accommodate variations in bioprocess set-up and design. Each step in CAR-T cell manufacturing may differ dependent on the kind of CAR-T cell therapy to be produced. As a hospital treats many different patients with different needs, the platform has to be suited to a multitude of ATMP products and allow for easy implementation of new ones (16). For instance, while genetic engineering has typically been achieved by viral transduction, more and more processes utilize non-viral transfection methods to transfer the CAR-DNA (17, 18). Therefore, a universally acceptable manufacturing platform should accommodate both viral and non-viral methods. Studies have also shown the efficacy of therapies with both CD4^+^ and CD8^+^ CAR-T cells (19). Currently, this is achieved by culturing both cell types separately and then combining them for the formulation of the therapy. To improve treatment efficacy without increasing process burden, a co-cultivation of CD4^+^ and CD8^+^ T cell populations is preferable to eliminate the need for two separate cell cultures running in parallel. Lastly, while some CAR-T cell therapies are cryopreserved before injection, some are held until product release and then directly transferred to the patient without prior freezing (20). Additionally, cryopeservation can thereby have a high impact on the outcome of the therapy (21). All of these different modes of operation need to be represented by the different hardware required for each individual process step, but also by a highly flexible software architecture allowing for these adaptions to different CAR-T products.

To automate autologous CAR-T cell therapy manufacturing, all required **hardware**, such as machines and devices, need to be combined in one integrated process pipeline. As depicted in Figure 2 the manufacturing plant consists of two sections–one for manufacturing and one for quality control. Both sections are automated and centrally controlled. The **manufacturing section** incorporates devices for cell washing, selection, electroporation, expansion, harvest, and formulation. Our approach, developed in AIDPATH, realizes CAR-T cell manufacturing by automating tubing-kit-based devices and interconnecting the tubing kits with sterile connectors and tube welding. Cells are automatically transferred to their next process step by connecting tubing assemblies. This significantly reduces the need for a large number of highly trained personnel to manufacture CAR-T cell products while also greatly reducing the direct interaction of personnel and product and thus risk of contamination.

A key element of the manufacturing section is the integration of a sophisticated perfusion bioreactor, which not only enables the much-needed co-cultivation of CD4^+^ and CD8^+^ CAR-T cells but is also equipped with various sensors allowing the deployment of AI-supported control strategies, which will be developed during the AIDPATH project.

As manufacturing protocols integrate feedback loops based on the outcome of analytical measurements and product release is highly dependent on the time required for analytical assays, the **quality control section** tightly integrates quality control processes. Therefore, the platform is designed to pass cell samples aseptically and automated from manufacturing to quality control. The quality control section features devices to conduct analytics for cell quantity, viability, identity, and characterization of the subpopulations present. A liquid handler, flow cytometer, and cell counter are integrated using a six-axis industrial robot. The quality control section is completed by integrating automation enabling solutions for common laboratory tasks such as container capping and de-capping of material restocking.

CAR-T cell manufacturing is a cost-intensive process, not only because of its labor-intensiveness but also because of the resources required. Current semi-automated devices rely on a one-device-per-patient approach, allowing for parallelization only by increasing device numbers. This leads to a linear increase in investment costs for parallelized CAR-T cell manufacturing. To make autologous cell therapy manufacturing economically more attractive, **parallelized production** without a linear increase of costs needs to be implemented. In our concept, this is achieved by increasing the number of cartridges in the bioreactor system. The longest step during manufacturing is expansion. Thus, parallelization can be achieved by exchanging each patient's incubation cartridge. As all manufacturing devices use closed tubing kits and these are replaced before the respective process step, the material of several patients may be processed in parallel without the risk of cross-contamination.

As CAR-T therapy is becoming a more established research area, the technologies applied for the generation of the therapy also evolve. Keeping up with technological advancements will require the integration of new devices. This is enabled by a **modular approach** to the manufacturing and quality control section. Integration of new devices for the manufacturing section is facilitated by the straightforward reconfiguration of the tubing assemblies, as these are equipped with standardized interfaces for sterile connection or welding. Retrofitting devices in the quality control section is also uncomplicated as the six-axis robotic handler features sufficient flexibility to provide different sample materials and carriers. Overall, this allows for **independence on manufacturers**, as various devices from different manufacturers can be integrated. Furthermore, although the platform is built for CAR-T cell manufacturing, the design is agnostic of cell type as the implemented technologies are also applied in other manufacturing processes. This makes the cell type manufactured on the platform in future developments flexible to a encompass variety of non-adherent, genetically engineered ATMPs.

For the **software** concept, two significant challenges arise. To enable end-to-end automation and eliminate all avoidable manual steps, a centralized execution, monitoring, and control of the entire process chain is required while integrating the various devices (22). Secondly, the AI models must be incorporated into the system to guarantee continuous data supply for model building and training and AI decision support for manufacturing control. Also, the software concept must consider the boundary conditions such as the modularity of the system, the volatile environment of ATMP manufacturing, and the regulatory landscape.

Currently, most devices in biotechnology are still being developed for manual operation. This makes automation difficult, as there are no interfaces for controlling the devices with external software or reading out data. Although standardized communication protocols such as OPC-UA and SiLA 2 are becoming increasingly important, they are not yet offered by most device manufacturers (23). Therefore, a middleware is needed for central control that generates a driver for each device. Each driver collects and sends data *via* a device's physical interface (e.g., USB, Ethernet) and then enables service-oriented communication with the **control software** *via* a standardized communication protocol. Here, the individual capabilities of the device (e.g., set temperature) are semantically described as a service. This semantically uniform description enables the flexible creation of protocols, integrating decisions (e.g., if the temperature is higher than X, then Y), and the low-effort integration of new devices (23, 24). A scheduling module ensures optimal machine utilization while scheduling all process steps including the parallelized manufacturing for the bioreactor cartridges. Digital batch records are generated automatically to avoid laborious manual documentation. In AIDPATH the software COPE is used and adapted to the requirements of the CAR-T cell process. The software was developed in several research projects for stem cell manufacturing (24, 25).

The making of AI models enables an intensive insight into biological processes and informed decision-making. However, they also require various patient data (e.g., age, gender, previous illnesses) and the manufacturing process (e.g., process and cell parameters, device information). These heterogeneous data sets are available in different qualities and formats and collected at different frequencies. Therefore, a **data management** framework must process all data using a standardized model, such as the OMOP Common Data Model (26), to ensure a general understanding and a straightforward analysis. Furthermore, data of different velocities must be integrated. Continuous data from sensors and devices in the manufacturing platform must be collected by a stream data platform (e.g., Apache Kafka) and made available to the AI models in aggregated form. Furthermore, a data storage platform is required that processes and stores batch data from patients and historical data sets. These data processing procedures and components form the foundation for the AI framework in which the various models are built, trained, and then deployed (27).

Another essential part of the automated AI-driven CAR-T cell manufacturing concept and the smart manufacturing hospital is the involvement of clinicians and technicians. Therefore, data can be integrated manually, automatically, and displayed in a user-specific way. Without expertise in software development, clinicians can create and customize process protocols using a drag-and-drop process creator. The decision support system, as part of the AI framework, transforms the results of the AI models into decisions and comprehensibly prepares them for human control and execution. This is brought together in a unified user interface, enabling centralized patient-specific process monitoring, data management, and manufacturing platform control.

From a regulatory point of view, our automated CAR-T cell manufacturing concept must comply with GMP and consider the GAMP guidelines (28). Also, the MDR [Medical Device Regulations (29)] will be taken into account. The smart manufacturing hospital's infrastructure will be designed to provide layers of in-depth cyber security and resilience to the manufacturing process. In case of a cyber incident, compromised segments are easily isolated to allow the infrastructure's continuous functioning.

## Artificial Intelligence in CAR-T Cell Manufacturing

AI can gain crucial process insights into the cell's characteristics and behavior. This offers a great advantage for adaptive control of the whole process and the creation of personalized process protocols. Furthermore, AI can support economic platform operation in the smart manufacturing hospital by optimizing manufacturing schedules and resource management. Therefore, AIDPATH will develop different AI applications along the CAR-T cell manufacturing and therapy process.

To get deeper process insights on the CAR-T cell process and understand how patient-specific characteristics influence it, a **digital twin** will track the product through the entire manufacturing process and perform simulations on the cell behavior. Based on these insights the control software can adaptively control the bioreactor in the time-consuming cell expansion process. From the recorded process data of the bioreactor, such as oxygen or lactose, the cells' status can be determined and possible expansion strategies simulated (30–32). The process data is thereby supplemented by metabolomics data, due to their promising characteristics for quality control in personalized therapy (33, 34).

The planning of the therapy requires solving a complex **resource allocation problem** under substantial uncertainty and with frequent replanning. The complexity comes from varying production times and the number of resources needed, such as medical equipment or intensive care beds. Additionally, the time frame of therapy has to be adjusted on a patient-by-patient basis during therapy, depending on the progression. While conventional optimization algorithms reach here their limits, reinforcement learning is a promising method with the ability to cope with these challenges. Also, **adaptive scheduling** can integrate the manufacturing process on the platform optimally into the overall therapy process (35). In therapy planning, decision support for the physician facilitates central decisions. This enables a personalized therapy for each patient independent of predetermined values.

Since all these AI applications are used in a sensitive environment, one of the crucial aspects is **trustworthiness** (36). In the AI application domain, trustworthiness can be made tangible by asking two guiding questions: How well can one specify the application's behavior? What risks are introduced by the application, and how can they be dealt with? The specificity can be divided into the main pillars explainability, robustness, and security. Here, trust is increased by explainable results, robust predictions, and safe behavior of the application outside the actual work domain. Risks that continue to exist can be quantified and dealt with by risk management methods (37, 38).

## Discussion

This perspective has discussed an initial concept of how automated AI-driven CAR-T cell manufacturing can be implemented directly at the point of care in a smart manufacturing hospital. It focuses on the engineering perspective and how hardware and software components must be designed to manufacture autologous CAR-T cells efficiently. The regulatory framework is a significant obstacle that needs to be overcome before a wide-scale deployment is possible. While the facility design has been GMP-complaint and GAMP guidelines for the software have been considered, there is a need for precise regulatory guidance from EMA and the FDA on using AI-driven manufacturing platforms. This refers to a validation of a reliable functioning of the AI algorithms and the assurance of trustworthiness (e.g., appropriate data quality and quantity for training, possibility for continouos training) (39, 40). Another issue is economic considerations. Reduced manual, cost-intensive handling steps are set against automation costs. Comparing a similar system for automated stem cell production shows the potential for overall cost reduction (41). However, a health economic assessment for this concept will be the subject of future development in AIDPATH. In addition to purely economic considerations, the supply situation for patients must, of course, also be considered. Due to the parallelization of the bioreactor, high scalability and high throughput can be aimed. The resulting shorter production and delivery times positively affect the number of patient treatments. Nevertheless, a discussion is needed to what extent centralized and decentralized CAR-T cell production can coexist in the future. Another point that is still up for discussion is the operator model. Although the automated processes and an intuitive user interface allow operation by non-highly qualified personnel, it is still unclear to what extent such a system can be operated by hospitals or external service providers, such as pharmaceutical companies. In particular, it must be taken into account that the operation and maintenance of the hardware, AI and IT infrastructure will result in new tasks for the operator.

All in all, this is a promising concept that needs to be adapted and further developed to the rapidly changing market of cell and gene therapies in the coming years (42). Here, the focus must be on the transferability of the concept because CAR-T cell therapy is only the beginning of ATMP development and deployment.

## Data Availability Statement

The original contributions presented in the study are included in the article/supplementary material, further inquiries can be directed to the corresponding author.

## Author Contributions

All authors listed have made a substantial, direct, and intellectual contribution to the work and approved it for publication.

## Funding

The paper was written within the framework of the EU project AIDPATH (grant agreement number 101016909). All mentioned colleagues/companies are part of AIDPATH and therefore received funding from the EU within the scope of AIDPATH.

## Conflict of Interest

JJ was employed by ORTEC BV. The remaining authors declare that the research was conducted in the absence of any commercial or financial relationships that could be construed as a potential conflict of interest. Companies Fraunhofer Institute for Cell Therapy and Immunology IZI, University College London, Foundation for Research and Technology (FORTH)-Hellas, SZTAKI, University Clinics Würzburg, Aglaris Cell SL, Sartorius Cell Genix GmbH, Fundació Clínic per a la Recerca Biomèdica, IRIS Technology Solutions, Red Alert Labs, Panaxea b.v., ORTEC b.v. were involved in the elaboration of the idea of the paper, the reviewing and the decision to submit the paper.

## Publisher's Note

All claims expressed in this article are solely those of the authors and do not necessarily represent those of their affiliated organizations, or those of the publisher, the editors and the reviewers. Any product that may be evaluated in this article, or claim that may be made by its manufacturer, is not guaranteed or endorsed by the publisher.
